# Functional and mutational analysis after radiation and cetuximab treatment on prostate carcinoma cell line DU145

**DOI:** 10.1186/s13014-021-01859-6

**Published:** 2021-07-28

**Authors:** Raik Schneider, Günther Gademann, Hans-Joachim Ochel, Karsten Neumann, Burkhard Jandrig, Peter Hass, Mathias Walke, Martin Schostak, Thomas Brunner, Frank Christoph

**Affiliations:** 1 Department of Radiotherapy, Otto-von-Guericke-University Magdeburg, University Hospital, Magdeburg, Germany; 2 Merck Serono Oncology, Darmstadt, Germany; 3 Department of Pathology, Hospital Dessau-Rosslau, Dessau, Germany; 4 Department of Urology, Otto-von-Guericke-University Magdeburg, University Hospital, Magdeburg, Germany; 5 Urology City West, Berlin, Germany

**Keywords:** Cetuximab, Radiation, DU145, Prostate cancer, Surviving fraction, Resistance mutations

## Abstract

**Background:**

Epidermal Growth Factor Receptor is often overexpressed in advanced prostate carcinoma. In-vitro-studies in prostate carcinoma cell line DU145 have demonstrated increased sensibility to radiation after cetuximab treatment, but clinical data are not sufficient to date.

**Methods:**

We analyzed effects of radiation and cetuximab in DU145 and A431 using proliferation, colony-forming-unit- and annexin-V-apoptosis-assays. Changes in protein expression of pEGFR and pERK1/2 after radiation and cetuximab treatment were analyzed. Using NGS we also investigated the impact of cetuximab long-term treatment.

**Results:**

Cell counts in DU145 were reduced by 44% after 4 Gy (*p* = 0.006) and 55% after 4 Gy and cetuximab (*p* < 0.001)*.* The surviving fraction (SF) was 0.69 after 2 Gy, 0.41 after 4 Gy and 0.15 after 6 Gy (each *p* < 0.001). Cetuximab treatment did not alter significantly growth reduction in 4 Gy radiated DU145 cells, *p* > 0.05 or SF, *p* > 0.05, but minor effects on apoptotic cell fraction in DU145 were detected. Using western blot, there were no detectable pEGFR and pERK1/2 protein signals after cetuximab treatment. No RAS mutation or HER2 amplification was detected, however a TP53 gen-mutation c.820G > T was found.

**Conclusions:**

Radiation inhibits cell-proliferation and colony-growth and induces apoptosis in DU145. Despite blocking MAP-Kinase-pathway using cetuximab, no significant radiation-sensitizing-effect was detected. Cetuximab treatment did not induce resistance-mutations. Further research must clarify which combination of anti-EGFR treatment strategies can increase radiation-sensitizing-effects.

## Background

Prostate carcinoma represents the most common cancer disease in men and affects 26% of all male cancer patients [1]. In locally confined tumor disease, radical prostatectomy or percutaneous radiation therapy is commonly applied [2, 3]. Radiated patients with higher risk for recurrence opt for adjuvant hormone ablation therapy with a Gonadotropin-Releasing-Hormon blocker [4].

The Epidermal Growth Factor Receptor is a 170 kD transmembraneous glycoprotein, its gene is located on chromosome 7p11.2 and it triggers particularly in epithelial tissues, mitosis, apoptosis, migration and cell differentiation [5, 6].

EGFR is overexpressed in nearly 50% of all cases of the prostate carcinomas [7]. EGFR protein expression levels increase with tumor stage and were highly correlated with hormone refractory status [8, 9].

In addition, signaling proteins of the MAP kinase network are known to be involved in the reactivation of androgen receptor function in prostate carcinoma cells in a cross talk manner [10].

Ionizing radiation leads to activation of proliferation and survival mechanisms via downstream EGFR-Mitogen-activated Protein Kinase and Phosphatidylinositol 3-kinases- Protein kinase B (Akt) signal pathways contributing to an increased resistance to the radiation.

Cetuximab, which targets the EGFR, can inhibit proliferation and cell cycle progression, thus activating apoptosis. It is also involved in the deactivation of specific survival mechanisms after radiation treatment. The efficacy of cetuximab as a so called “radiosensitizer” depends on the accumulation of mutations in gene of downstream signal transduction protein kinases of the EGFR-pathway (e.g. RAS and RAF mutations) and certain mutations of the EGFR tyrosine kinase domain o﻿r extracellular domain [11]. Mutations in BRAF codon 600 and KRAS codon 12 and 13 were found in 10.2% and 73% of prostate adenocarcinomas respectively and have a higher propensity for higher PSA, Gleason score and tumor stages with BRAFV600 mutations [12]. A common genetic aberration that increases during the course of transformation to more malignant prostate carcinomas is a deletion of exons 2–7 of the extracellular domain of EGFR, resulting in constitutively active EGFR variant III (EGFRvIII) [13].

Recent data have shown that after cetuximab treatment, the repair of radiation induced DNA double-strand breaks is suppressed by a reduced cross-regulated EGFR DNA-PKcs complex in the nucleus. Radiosensitizing by cetuximab therefore affects the EGFR-function by slowing down DNA repair and enhancing reproductive cell death in tumor cells [14].

The activation of inhibitory pathways of cell proliferation after cetuximab treatment in prostate carcinoma cell lines LNCaP, PC3 and DU145 depends upon androgen receptor status, cell line specific molecular profiles and the occurrence of specific resistances [15]. PC3 is a PTEN-negative prostate carcinoma cell line with constitutive activation of the PI3K-Akt ﻿pathway.﻿ LNCaP is an androgen receptor-positive and androgen-dependent growing prostate carcinoma cell line. In contrast, the cell line DU145 is AR-positive but androgen-independent growing, EGFR expressing, and also moderately radiosensitive. Therefore, an additional antiproliferative effect was only expected in the DU145 prostate carcinoma cell line by cetuximab [16]. In a report by Wagener et al. and Liu et al. a moderate suppressive effect on proliferation rate by either radiation or cetuximab alone has been observed in DU 145 prostate carcinoma cells which was more pronounced in combination [17, 18].

To show differences in cetuximab effects after irradiation in DU145, we used the cell line A431 as a positive control. A431 is a human skin epidermoid cell line with high EGFR overexpression and radiosensitivity and cetuximab sensitivity.

In the present study, we aimed at investigating the radiosensitizing effect of cetuximab in the prostate carcinoma cell line﻿ DU145 using various methods to identify specific resistance mutations after cetuximab long-term application. We also evaluated the consistency of the effects observed, with regard to future treatment strategies that combination therapy could provide in advanced prostate carcinoma [19].

## Methods

### Characterization and quantification of tumor cells

DU145—a human, androgen-independent prostate carcinoma cell line and A431—a human, epithelial, EGFR-overexpressing epidermoid carcinoma cell line was obtained from Leibniz-Institute German Collection of Microorganisms and Cell Cultures GmbH, Braunschweig.

The rate of cell growth in cells per ml was monitored regularly using the automatically Scepter™ cell count pipette. Cells were cultivated in 10 ml Roswell Park Memorial Institute 1640 medium 1640 + 10% Fetal Bovine Serum and used for experiments in their exponential growth phase.

### Cetuximab and radiation treatment

Cells were cultivated in 100 nM cetuximab- (Merck, Darmstadt) containing cell culture medium for four hours prior to radiation and permanently in cetuximab-containing cell culture medium after radiation.

Standardized radiation doses were applied using the Gulmay X-ray therapy unit D3225 using a radiation stay time table (radiation stay time based on farmer chamber measurement 30013-0415 on 31.05.2012/27.06.2012). The desired dosage (Gy) was achieved by defining radiation amount per time unit according to radiation stay time table.

### Proliferation and colony forming assay

Cells of the tumor cell lines were harvested and diluted in cell culture medium for counting. 20,000 cells per 10 ml dish were seeded and adhere in duplicates per measurement 48 h prior to the proliferation test.

On Day 3, cetuximab was added four hours prior to radiation (4 Gy, once). Culture medium, which was renewed one day after radiation, contained cetuximab for the whole proliferation period.

Cells of radiation alone, cetuximab and radiation + cetuximab group, were counted in exact 24-h intervals in three independent measurements for the following 8 days.

In preparation for the colony forming trials, an appropriate number of 5 × 10^5^ – 1 × 10^6^ cells were cultivated overnight and treated in the same procedure as in proliferation assay.

After radiation, the cell layer was washed and dissociated by trypsinization. At an initial density of 10,000 to 20,000 cells/ml the optimal plating concentrations for building cell colonies could be calculated and either 500 or 1,000 cells were seeded in two replicates per dose and a no treated control group. The treatment of the tumor cells with cetuximab was maintained throughout the colony-building period by refreshing cetuximab containing medium. After twelve days, the colonies were stained and quantified.

### Apoptosis detection with annexin V

To determine the proportion of living cells in apoptosis after radiation ± cetuximab a Fluorescence-Activated-Cell-Sorting system BD Canto™ II after staining with annexin V-allophycocyanin with Dead-Cell Apoptosis Kit (Life Technologies) and SYTOX® Green as live/dead vitality staining where used.

### Western-blot experiments

Western-blot experiments served to verify the EGFR on the protein level, its phosphorylated form, and its activated effector molecule pERK1/2. Prior to the Western-blot experiments, the cells were treated according to protocol and/or stimulated with EGF 10 min prior to cell lysis.

After blotting, primary antibodies anti-EGFR clone H9B4, anti-phospho-EGFR (pY1173), (Invitrogen™), phospho-p44 + p42 MAPK, (pThr 202 + pTyr 204, ERK1/2), (ThermoFisher Scientific) were added which was followed by the incubation with secondary, HRP-conjugated anti-mouse and anti-rabbit IgG whole antibodies (GE Healthcare).

After incubation in 1:1 Super-Signal-West® Pico-Stable-Peroxide and Luminol/Enhancer-Solution for one minute, the photograph was developed in Agfa CURIX 60 image processor.

### Molecular genetic testing of cetuximab resistance

To verify secondary cetuximab-induced resistance mutations, DU145 and A431 cells were incubated for up to nine months with a monthly increasing cetuximab concentration that progressed from 5 to 50 and from month four with 100 µg/ml cetuximab.

DNA preparation was done with QIAamp® DNA-Mini-Kit 50 (Qiagen®). DNA concentration in the samples was determined through measuring optical density at 230 nm in ng/µl in the NanoDrop® ND 1000 photometer.

After amplification and labelling, fifteen target genes from the DNA-libraries of samples from treated and untreated cells were sequenced with a TruSight® Tumor 15 Panel and next generation sequencing (NGS, Illumina®) technologies (Table 1).

**Table 1:** TrueSight Tumor 15 panel—Target genes NGS investigated for secondary resistance alterations after long-term treatment with cetuximab in DU145 and A431 cells

TruSight® Tumor 15 Gene	Target of Region
*AKT1*	E17K
*BRAF*	V600E/K/R/M
*EGFR*	Focal Amplification, Exon 19, Exon 20, G719A, Exon 21 (L858R), L861Q, S7681, T790M
*ERBB2*	Focal Amplification, p.E770_A771insAYVM (equivalent to p.A775_G776insYVMA)
*FOXL2*	C134W
*GNA11*	Q209L
*GNAQ*	Q209L
*KIT*	Exons 8,9,10,11,13,14,17,18
*KRAS*	Codons 12,13,59,61,117,146
*MET*	Focal Amplification
*NRAS*	Codons 12,13,59,61,117,146
*PDGFRA*	Exons 12,14,18
*PIK3CA*	Exons 9,20
*RET*	M918T
*TP53*	Full CDS

The obtained sequences were aligned and mapped to a reference sequence matrix. Potential deviations from reference sequences were analyzed using Illumina-Variant-Studio-Data-Analysis-Software.

### Statistical analysis

Pairwise comparison between treatment group and control group in proliferation assay (cell count) and colony forming assay (survival fraction) in both cell lines was analyzed using Kruskal–Wallis-Test. To compare the differences in the irradiation and the irradiation + cetuximab group in both cell lines the Mann–Whitney-*U*-Test was used.

## Results

**Fig. 1:**
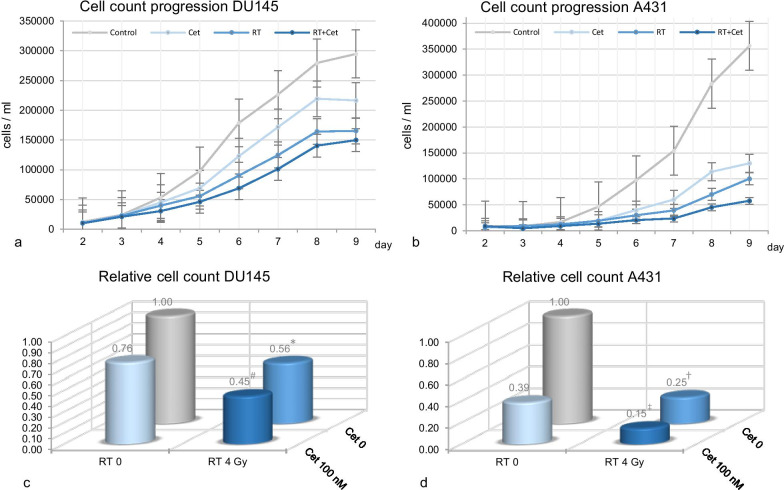
Proliferation assay—Cell count-based growth curve from day 2 to day 9 with and without treatment, DU145 (**a**) and A431(**b**); Proliferation assay—relative cell count day 7 after radiation 4 Gy ± cetuximab 100 nM DU145 (**c**) significant reduction after radiation *p = 0.006 and radiation + cetuximab ^#^*p* < 0.001, no significant after cetuximab ^ƚ^*p* = 0.35; no differences between radiation and radiation + cetuximab *p* > 0.05, radiation and cetuximab *p* = 0.73; significant between cetuximab and radiation + cetuximab *p* = 0.031 and A431 (**﻿d**) significant reduction after radiation ^†^*p* = 0.02 and radiation + cetuximab ^‡^*p* < 0.001, after cetuximab ^ƚƚ^*p* = 0.09; no differences between radiation and radiation + cetuximab *p* > 0.05, radiation and cetuximab *p* > 0.05 and between cetuximab and radiation + cetuximab *p* = 0.53; cetuximab 100 nM on day 2 and 3, radiation 4 Gy on day 3; (RT = radiation, Cet = cetuximab)

The daily growth rate from a starting cell count of 20,000 sown, untreated cells in cell line DU145 and 10,000 cells in A431 was significant in the observed period of nine days. From Day 7 we could determine a difference cell counts in DU145 between control and radiation at 4 Gy as well as control and radiation + cetuximab treated cells; there was no difference between control and cells treated with cetuximab only. Relative reduction in cell count in DU145 on Day 7 in comparison to control was 55% after combined treatment, *p* < 0.001, 44% after radiation with 4 Gy, *p* = 0.006 and 24% after cetuximab treatment only, *p* = 0.35. On day 7, no significant differences in cell number were observed between irradiation vs. irradiation + cetuximab, *p* > 0.05 and irradiation vs. cetuximab, *p* = 0.73, while there was a significant difference between cetuximab vs. irradiation + cetuximab, *p* = 0.031. The relative reduction in cell count in A431 on Day 7 was 85% after combined treatment, *p* < 0.001, 75% after radiation with 4 Gy, *p* = 0.02, and 61% after cetuximab treatment only, *p* = 0.09. On day 7, no significant differences in cell count were observed in A431 in the different treatment groups (Fig. 1a–d).

Comparing DU145 and A431 cell lines to each other at day 7, a significantly greater decrease in cell proliferation was found in A431 after irradiation (*p* < 0.001), cetuximab (*p* = 0.003) and irradiation + cetuximab (*p* < 0.001).

We determined the average plating efficiency (PE) to be 0.54 in cell line DU145 and 0.33 in cell line A431. After radiation at 2 Gy the average surviving fraction (SF) was 0.69 (DU145) and 0.54 (A431), at 4 Gy it was 0.41 and 0.25 respectively, at 6 Gy it was 0.15 and 0.11. Comparing the control group with the radiated groups and the differently dosed groups with each other showed that the decline of the SF was significant in each case (*p* < 0.0001).

**Fig. 2:**
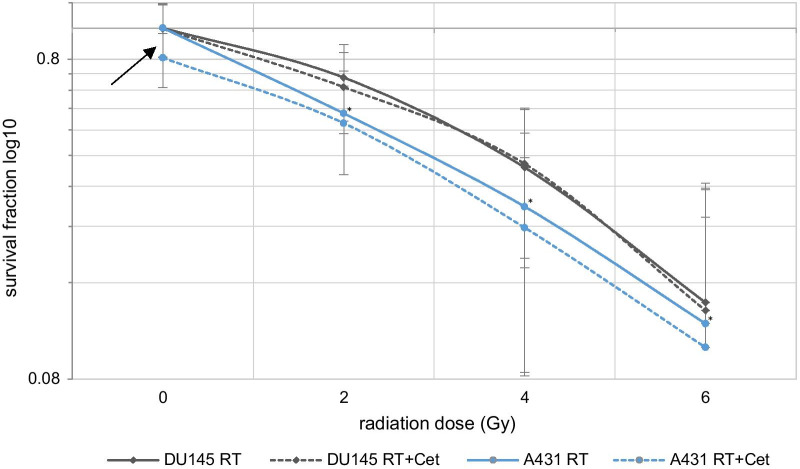
Colony formation assay DU145 and A431—Logarithmic depiction of the impact of radiation ± cetuximab on SF, *p < 0.001 in relation to radiation doses—2, 4 and 6 Gy between cell lines, the black arrow indicates the effect of cetuximab on the SF only in A431, (RT = radiation, Cet = cetuximab, SF = survival fraction)

Cetuximab had no (DU145) or only low (A431) impact on the decline of SF after radiation at 4 Gy. Average SF was 0.37 and 0.28 after radiation at 4 Gy; compared to radiation + cetuximab SF was 0.37 (*p* = 0.71) and 0.24 (*p* = 0.09) respectively. Cetuximab treatment alone resulted only in cell line A431 in a decline of the average SF to 0.81, *p* < 0.001 (Fig. 2).

Quantifying Apoptosis by FACS-analyses, radiated DU145 cells with 0 up to 6 Gy showed no dose-dependent increase of apoptotic cells in comparison to the untreated control group. Additional cetuximab treatment over all radiation doses had a minimal impact on the apoptotic rate in DU145 cells.

**Fig. 3:**
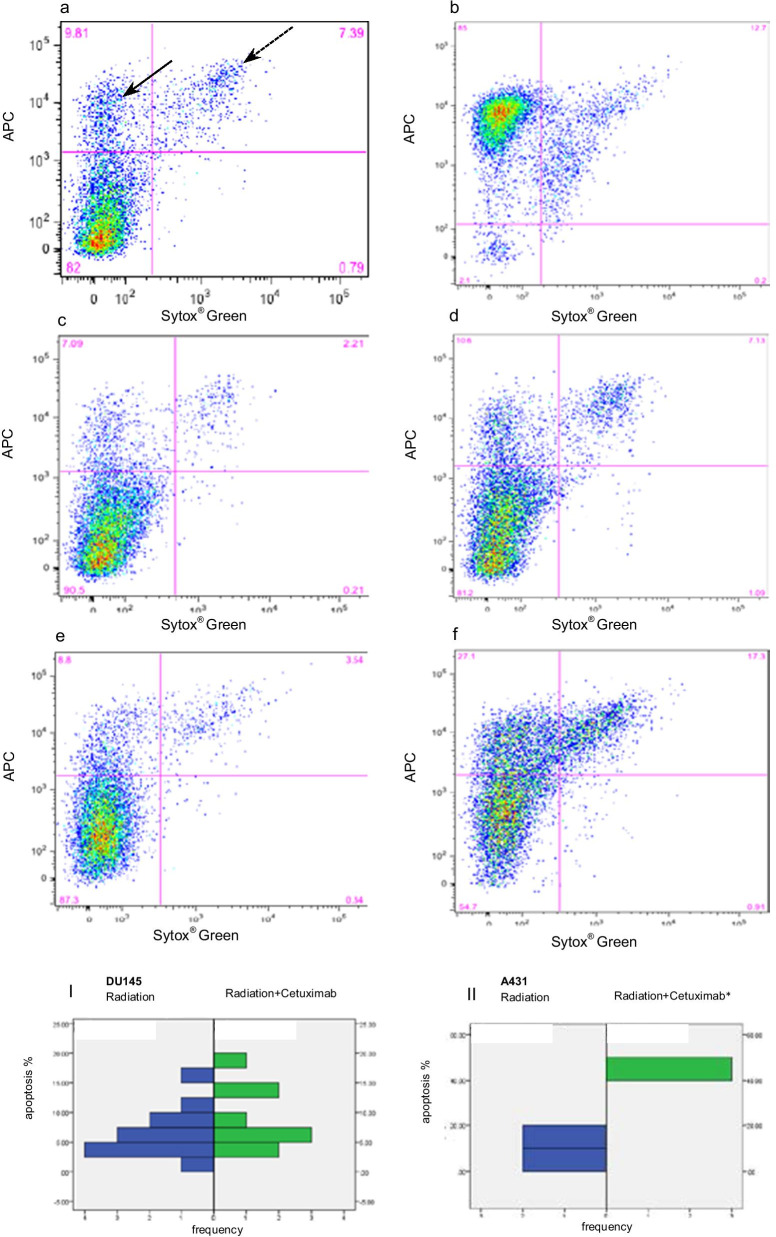
DU145-A431 apoptosis FACS-assay, APC-Annexin V/Sytox® Green staining, **a** without treatment, **b** 2 mM H_2_O_2_ treatment, **c** DU145 RT 4 Gy, **d** A431 RT 4 Gy, **e** DU145 RT 4 Gy + Cet 100 nM, **f** A431 RT 4 Gy + Cet; I/II) Mann–whitney-u-test, apoptosis-comparison between treatments, II*p = 0.057, (black arrow shows apoptosis fraction, dotted black arrow shows late-apoptosis fraction)

In cell line A431 there was a notable increase of the apoptotic fraction after radiation, in comparison to control. The average apoptotic rate during a combined treatment with radiation and cetuximab was 45% and showed in comparison with the 11% rate during radiation treatment alone, an increasing trend (*p* = 0.057, Fig. 3). Comparing the apoptotic rate in both cell lines regarding all treatments (early apoptosis: *p* = 0.006, late apoptosis: *p* = 0.043), radiation alone (only late apoptosis: *p* = 0.018), as well as combined treatment (only early apoptosis: *p* = 0.009) a significantly higher apoptotic rate was observed in A431.

**Fig. 4:**
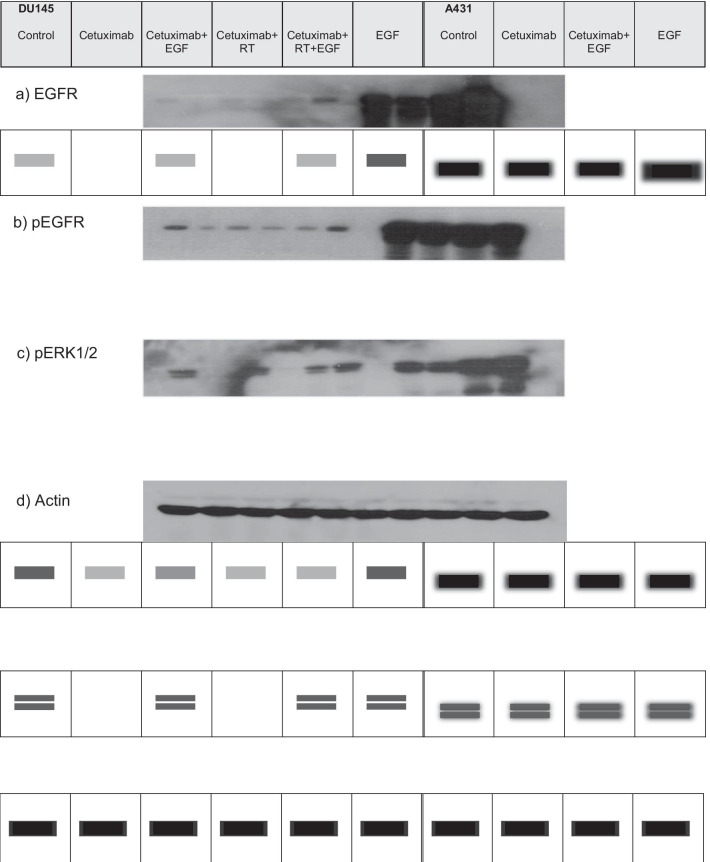
Western-blot analysis—**a** left side: cell line DU145—EGFR-protein bands after radiation + cetuximab (± EGF), right side: cell line A431-EGFR protein bands after cetuximab (± EGF), **b** phosphoEGFR-protein bands, **c** p44/p42 Erk1/Erk2-protein bands, **d** Actin, RT = radiation; Note: the table below the respective photo is the associated abstract drawing of the corresponding protein bands

The protein expression of the EGFR in DU145 is reduced after cetuximab treatment. This was also observed after the combined treatment. The expression of the EGFR was pronounced in cell line A431 and was influenced by cetuximab only to a small degree. In DU145, the protein band of the pEGFR was completely suppressed after cetuximab, independent from radiation treatment. EGF (re)induced a weak signal. In A431, the pEGFR protein signal was considerably weakened after cetuximab treatment. After additional EGF treatment, a strong signal was visible. The activated form pERK1/2 was completely suppressed in DU145 after cetuximab treatment independent from radiation. In cell line A431 the suppression is not complete and EGF stimulates the phosphorylated ERK1/2 protein as well (Fig. 4).

The long-term treatment of DU145 and A431 cells with cetuximab did not cause secondary mutations in the KRAS, NRAS- or BRAF-V600 genes. Likewise, no modifications were detected in Exon 9 and 20 of the PI3KCA nor typical amplifications in the HER2-receptor gene.

In the TP53 gene we detected different point mutations for both cell lines that were unrelated to the long-term cetuximab treatment. The TP53 gene in DU145 expressed the mutation c.820G>T with amino acid replacement p.Val274Phe with a frequency of 65%. In A431 it expressed the mutation c.818G>A with p.Arg273His and a frequency of 100% (Table 2).

**Table 2:** Frequency of detected nuclein base- and amino acid change in the TP53-gene in cell line DU145 and A431 respective ± cetuximab (l)^†^, ^†^long-term treatment

Zelllinie	Gene	Amino Acid Change	Variant Type	Nucleotide Change	Variant Frequency
DU145	*TP53*	p.Val274Phe	missense variant	c.820G > T	0.641
DU145 + Cetuximab (l)^†^	*TP53*	p.Val274Phe	missense variant	c.820G > T	0.652
A431	*TP53*	p.Arg273His	missense variant	c.818G > A	0.994
A431 + Cetuximab (l)^†^	*TP53*	p.Arg273His	missense variant	c.818G > A	0.996

**Fig. 5:**
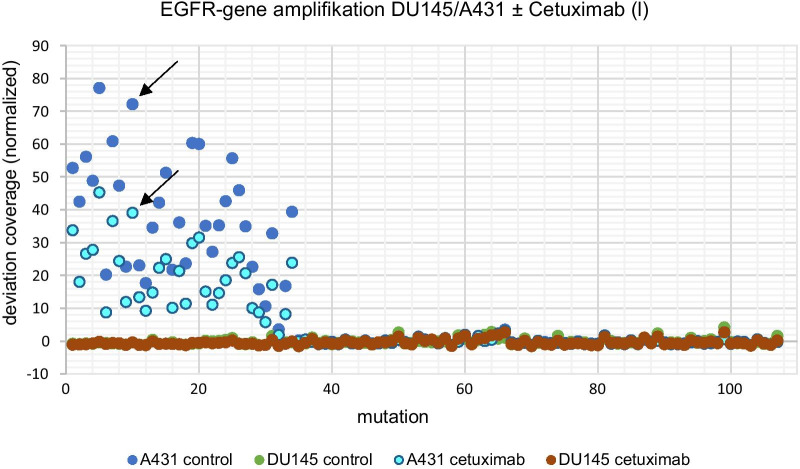
Mutation analysis—EGFR-gene amplification in chromosome 7 of cell lines DU145 and A431 ± cetuximab (l), the arrows exemplary show the difference of the deviation factor of the normal value coverage in A431 without (dark blue dots) and after cetuximab (l) treatment (light blue dots) *p* < 0.001; l = long-term treatment

Only A431 cells showed a notable amplification of the EGFR gene. The sequencing rate for untreated A431 cells increased up to 77-fold in comparison to the standard value. After long-term cetuximab treatment the sequencing rate was reduced significantly to half the value of the untreated sample (*p* < 0.001, Fig. 5).

**Fig. 6:**
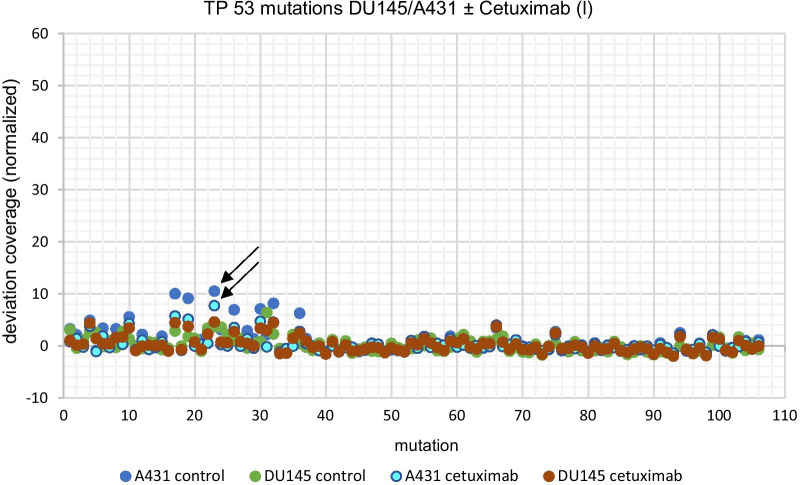
Mutation analysis—missense mutation TP53 gene in chromosome 17 of cell lines DU145 and A431 ± cetuximab (l), the arrows exemplary show the deviation factor of the normal value coverage in A431 without (dark blue dots) and after cetuximab (l) treatment (light blue dots) *p* = 0.015; l = long-term treatment

We detected significantly lower TP53 mutation frequencies after long-term cetuximab treatment compared to the untreated control group (*p* = 0.015, Fig. 6).

## Discussion

In our study we have shown that cell growth in DU 145 prostate ﻿cancer cell line is significantly reduced when radiation and cetuximab is applied in combination therapy but not after cetuximab treatment alone. In contrast, cell growth in cell line A431 was significantly reduced in all treatment branches also with cetuximab alone. In A431, the antiproliferative effect was most effective after combined treatment of radiation and cetuximab. These results are in accordance with the results presented by Dhupkar et al., who noted a more significant suppression of cell proliferation following cetuximab treatment in A431 than in DU145 [15]. However, a significant additive or synergistic effect of cetuximab to radiation was not observed in either DU145 or A431. DU145 cells appeared to be more radiation resistant and less susceptible to cetuximab in a proliferation assay. Recent data explain this incomplete suppression by cetuximab with an increased formation of heterodimers between EGFR and Human Epidermal Growth Factor Receptor 2 (HER2), which are formed alongside EGFR homodimers after radiation [20]. EGFR-specific ligands such as amphiregulin and epiregulin are particularly upregulated in hormone-refractory prostate carcinoma cells [21]. Epiregulin can activate cell proliferation by stimulating not only the EGFR but also heterodimer complexes of the HER-family. In addition, the formation of HER2/HER3-heterodimers in combination with the upregulation of HER3’s physiological ligand neuregulin-1 alternatively activates the PI3K/Akt-signaling pathway [22].

We found a dose dependent suppression of colony formation by radiation in both cell lines, but the addition of cetuximab did not significantly increase its effect in DU145 prostate cancer cell line. In 2010, Liu et al. found that the relative biological effectiveness (RBE = SF_rad_/SF_rad+cet_) of treated DU145 cells with 2 Gy irradiation ± cetuximab was 1.39, in contrast to our calculation for DU145 RBE at 4 Gy was 1.02 [18]. However, the suppression of colony formation in A431 cells was significantly increased by cetuximab alone treatment (SF 0.81, *p* < 0.001). Wagener et al. showed that cetuximab increases the susceptibility of DU145 cells to radiation treatment, but the additional effect was not significant [17]. ﻿These results could not be fully verified in the colony formation assay carried out in this study. We suggest a high variability of the proliferation regulation in androgen non-responsive DU145 cells through alternative signaling pathways such as PIK3-AKT pathway.

The apoptosis fraction of living DU145 cells using FACS analysis was not significantly increased after radiation or combined treatment. Radiated A431 cells, however, showed in both tests pronounced apoptosis, which was further increased by additional cetuximab treatment. Brown et al. highlighted that there is no sufficient evidence for the correlation between the extent of the apoptosis and the clinical response of solid tumors of epithelial origin [23]. Non-apoptotic pathways such as necrosis, mitotic catastrophe, or senescence are often more important factors in determining the programmed cell death. While maintaining their metabolic functions, senescent cells are incapable of completing the cell cycle [24].

The functional loss of one or both alleles of the TP53 gene due to missense mutations for DU145 have been described by Lehmann et al. [25]. TP53 missense mutations in the prostate carcinoma cell split up early into a cell type that undergoes complete functional loss of tumor suppression into a “dominant negative” phenotype. Consequently, G2/M arrest in the cell cycle will be absent, leading to further mutations, genetic instability and reduction of repair capacity. In the clinical setting, an increased radiation resistance and degeneration of prostate carcinoma cells can be observed in these patients [26]. With TrueSight® NGS technology, we identified the c.820G>T mutation with a frequency of 65%, leading to a functional loss of TP53 in the majority of cells. This mutation was visible in untreated cells as well as in those undergoing long-term cetuximab treatment.

Kumar et al. have shown that EGFR amplifications can occur in benign prostatic hyperplasia as well as in carcinoma cells [27]. However, in our analysis, no gene amplification of the EGFR, the HER2, or the c-Met has been observed in cetuximab treated DU﻿﻿﻿﻿145 prostate carcinoma cells. By contrast, the high level of EGFR amplifications in cell line A431, was reduced after long-term treatment with cetuximab. We predict cells with higher amount of gene copies and a high EGFR expression level to undergo apoptosis during long-term cetuximab treatment, resulting in lower level of amplicons in the remaining cell population.

EGFR protein signals were downregulated irrespective of radiation treatment in cetuximab treated DU145 cells and phosphorylation at Tyr-1173 binding site was suppressed. This down-regulation of total EGFR suggests binding of cetuximab to EGFR and its possible internalization and is in agreement with the findings of Dupkar et al. [15]. Cetuximab also suppressed downstream p44/p42 Erk1/Erk2 expression entirely in DU145 and partially in A431. This complete block of the signaling cascade contrasts the weak suppression of cell proliferation and apoptosis induction which was observed in DU145 cells. We hypothesize that DU145 cells employ alternative signaling pathways due to the EGFR/HER2 activity. Our results for the DU145 cell line can be considered preliminary. It would therefore be interesting to test further radiation-sensitizing substances, including for the prostate cancer cell lines LNCaP and PC3.

There is clinical evidence for a molecular-pathological shift during the progression of prostate cancer from hormone-naive to hormone-independent stage. The loss of radiosensitivity and its implication on presentation and activation of the EGFR during progression should lead to new treatment strategies for prostate cancer using targeted therapies [28]. It has already been applied in the treatment of head and neck cancer showing an improvement of tumor-specific survival following additional cetuximab treatment during radiation therapy [29]. However, it cannot easily be transferred to the situation in advanced prostate carcinoma [30].

Very recently, the PARP-inhibitor Olaparib has proven to have antitumor activity in vitro and in vivo in prostate cancer. Fenerty et al. have shown that addition of cetuximab to EGFR+, BRCA-mutated prostate cancer cell line 22RV1 or BRCA-wildtype cell line DU145 treated with olaparib increases Antibody Dependent Cell-mediated Cytotoxicity by natural killer cell lysis up to 2.8-fold resp. 1.7-fold after 36 h as compared to olaparib-only treatment [31]. Furthermore, immune checkpoint inhibitors have emerged as potential therapeutic partners, the EGFR antibody cetuximab could be helpful by triggering immunogenic cell death [32].

## Conclusions

Radiation inhibits cell-proliferation and colony-growth and induces apoptosis in DU145. Despite the blocking of the EGFR-MAP-Kinase pathway, an additive or synergistic effect of radiation plus cetuximab treatment could not be verified. Cetuximab long-term treatment did not cause typical resistance mutations in DU145. It remains to be proven, whether a combined approach of cetuximab with new complementary chemo- or immunotherapeutics will improve in vitro or in vivo inhibitory growth effects on prostate cancer cells following radiotherapy.

## Data Availability

The datasets used and/or analysed during the current study are available from the corresponding author on reasonable request.

## Abbreviations

BRAF: B-Raf proto-oncogene, serine/threonine kinases
BRCA: BReast cancer
EGFR: Epidermal growth factor receptor
EGF: Epidermal growth factor
ERK: Extracellular-signal regulated kinases
FACS: Fluorescence-activated-cell-sorting system
HER: Human epidermal growth factor receptor
MAPK: Mitogen-activated protein kinase
PARP: Poly (ADP-ribose)-polymerase
PE: Plating efficiency
PI3K: Phosphatidylinositol 3-kinases
PKcs: Proteinkinase catalytic subunit
PTEN: Phosphatase and tensine homologue deleted on chromosome 10
RAS: Rat sarcoma
RBE: Relative biological effectiveness
SF: Surviving fraction
